# Follicular Lymphoma in Chile in the Adult Public Cancer Program: The Impact of Chemoimmunotherapy

**DOI:** 10.1002/cnr2.2126

**Published:** 2024-09-22

**Authors:** María Elena Cabrera, Camila Peña, Valeska Vega, Hernán Rojas, Alvaro Pizarro, Christine Rojas, Susana Calderon, Jacqueline Oliva, Cecilia Hales, Bernardita Rojas, Marvila Intriago, Marisa Capurro, M. Luisa Gonzalez, Jorge J. Castillo

**Affiliations:** ^1^ Hospital del Salvador Universidad de Chile Santiago Chile; ^2^ Hospital del Salvador Providencia Chile; ^3^ Hospital San Juan de Dios Santiago Chile; ^4^ Hospital Sótero del Río Santiago Chile; ^5^ Hospital San Borja Arriarán Santiago Chile; ^6^ Hospital Gustavo Fricke Valparaíso Chile; ^7^ Hospital Regional de Valdivia Valdivia Chile; ^8^ Hospital Las Higueras de Talcahuano Talcahuano Chile; ^9^ Hospital Juan Noé Arica Chile; ^10^ Hospital Carlos van Buren Valparaíso Chile; ^11^ Hospital Regional de La Serena La Serena Chile; ^12^ Hospital Dr. Hernan Henriquez Aravena Temuco Chile; ^13^ Hospital Regional de Osorno Osorno Chile; ^14^ Division of Hematological Malignancies Dana‐Farber Cancer Institute, Harvard Medical School Boston Massachusetts USA

**Keywords:** FLIPI, follicular lymphoma, POD24, R‐CHOP, R‐COP

## Abstract

**Background:**

Follicular lymphoma (FL) is the most common indolent non‐Hodgkin lymphoma (NHL) in the United States and Europe. However, data on FL from Latin America are scant.

**Aims:**

This study aims at better understand the clinical features, treatment patterns and outcomes of patients with FL in Chile. Of special interest was to evaluate POD24 as an adverse marker.

**Methods and Results:**

We collected retrospective data from 722 patients 15 years or older diagnosed with FL and treated in 17 cancer centers in Chile between 2000 and 2019. Time to first treatment (TTFT), progression‐free survival (PFS) and overall survival (OS) were estimated using the Kaplan–Meier method. Cox proportional‐hazard regression models were fitted to investigate prognostic factor. The median age at diagnosis was 62 with a female predominance (63%); 73% of patients had advance stage disease and 68% had bone marrow involvement; 63% had intermediate or high FLIPI scores. The 1‐year TTFT rate was 96%, and 30% of patients received chemoimmunotherapy. Adding rituximab to chemotherapy was associated with a higher complete response (69% vs. 60%; *p* < 0.001) and superior median OS (16 vs. 8 years; *p* < 0.001). Patients who experience POD24 had an inferior median OS (2.4 vs. 15 years).

**Conclusion:**

Our study shows a female predominance in patients with FL in Chile and confirms superior response and survival outcomes with adding rituximab to chemotherapy. Our study also confirms a poor OS in patients who experience POD24.

## 1 Introduction

Follicular lymphoma (FL) is the second most common non‐Hodgkin lymphoma (NHL) in the Western world and accounts for 25%–40% of all cases [1, 2]. In Chile, FL accounts for 25% of lymphoma cases [3]. FL is an indolent, incurable neoplasia characterized by continual relapses [4]. Several prognostic indexes in current use remain inaccurate in identifying those patients at risk of early progression, relapse, or histological transformation. Follicular Lymphoma International Index (FLIPI) and FLIPI2 are commonly used [5, 6]. Other prognostic models using clinical and genetic variables have been evaluated, such as the GELF (Groupe d'Etude des Lymphomes Folliculaires) criteria, the PRIMA‐PI and the m7‐FLIPI [7]. Patients who progressed within 2 years (POD24) have an inferior prognosis [8]. POD24 has emerged as an important prognostic marker in FL.

Observation continues to be appropriate for asymptomatic patients with low tumor burden and no cytopenia. The combination of anti‐CD20 monoclonal antibodies, such as rituximab, with chemotherapy (CT) regimens like cyclophosphamide, doxorubicin, vincristine, and prednisone (CHOP), bendamustine, or cyclophosphamide, vincristine, and prednisone (CVP/COP) has been shown, in multiple studies, to result in improved overall response rates, longer duration of response, increased progression‐free survival (PFS), and overall survival (OS) compared to CT alone [9, 10, 11, 12, 13, 14, 15]. Rituximab maintenance improved PFS but not OS [16, 17]. The outcome of FL patients is quite variable, going from those who progress and die early to those who have an indolent course with prolonged remissions [4, 7, 18, 19].

Data on FL from Latin America and Chile are lacking. The Adult Cancer Nacional Program (PANDA) of the Ministry of Health has financed lymphoma treatments since 1988, and since 2007, rituximab has been routinely administered along with CT.

This study analyzed the demographic, clinical, and survival characteristics of patients with FL in Chile, focusing on determining the impact of rituximab on the outcomes of FL patients.

## 2 Patients and Methods

### 2.1 Patients

Patients with FL ≥15 years old, diagnosed and treated in 17 cancer centers in Chile, between 2000 and 2019, were included. The patients were registered prospectively in a database at the Cancer Unit of the Ministry of Health. The Scientific Ethics Committee of *Servicio de Salud Metropolitano Oriente* in Santiago, Chile, approved the study. All patients signed informed consent to be treated and have their data collected.

The histopathological diagnosis was centralized and performed by a reference pathologist. The staging workup followed the National Ministerial Guidelines, updated according to the literature evidence. HIV‐positive cases were included. Ann Arbor clinical staging was used. Patients were stratified according to the standard FLIPI and were divided into three risk groups: low (0–1 factor), intermediate (2), and high (3–5).

### 2.2 Treatment

Before introducing rituximab, patients received CHOP, COP, CHOP/COP, chlorambucil (CL), or radiotherapy (RT) alone. When rituximab was introduced in 2007, the therapy was R‐CHOP or R‐COP. The response was evaluated after 3 cycles with computed tomography (CT) scans. Response criteria were defined according to the International Consensus for Lymphoma [20]. Refractory or relapsed cases got mainly etoposide, cisplatin, methylprednisolone, cytarabine (ESHAP), fludarabine, cyclophosphamide, mitoxantrone (FCM), R‐CHOP, R‐bendamustine, or CL. Autologous stem cell transplant (SCT) was not performed, nor rituximab maintenance.

### 2.3 Definition of POD24

POD24 was defined as a progressive disease after stable disease or an initial response but later relapse/progression, or transformation, within 24 months after starting first‐line systemic treatment [8].

### 2.4 Follow‐Up

Patients had a clinical evaluation every 4 months during the first year, every 6 months from the second to fifth year, and annually thereafter. CT scans were performed as clinically indicated. The cause of death was taken from Civil Registry Death Certificates.

### 2.5 Statistical Analysis

Clinical characteristics and response to treatment were compared between pre‐ and post‐rituximab eras if they received at least one cycle of chemotherapy. Patients' characteristics are presented using descriptive statistics. Median time to first treatment (TTFT) was defined as the time from diagnosis to first treatment. OS was defined as the time from the date of diagnosis until death or last follow‐up. PFS could not be calculated since we had no such data in all cases. OS was calculated using the Kaplan–Meier method for incomplete observations and compared using the log‐rank test. Univariate and multivariate Cox proportional‐hazard regression models for OS were fit using a stepwise approach to investigate prognostic factors. Since the FLIPI score includes several investigated factors, such as age, LDH levels, hemoglobin levels, and stage, FLIPI was not included in the multivariate analysis to avoid overfitting the model. Missing values were as follows: ECOG (*n* = 67; 9%), hemoglobin (*n* = 110; 15%), LDH (*n* = 86; 12%), and FLIPI (*n* = 80; 11%). Missing data were handled using listwise deletion. Statistical calculations and graphs were obtained using STATA version 17 (StataCorp, College Station, TX, USA). *p*‐values <0.05 were considered statistically significant.

## 3 Results

### 3.1 Patients

**FIGURE 1:**
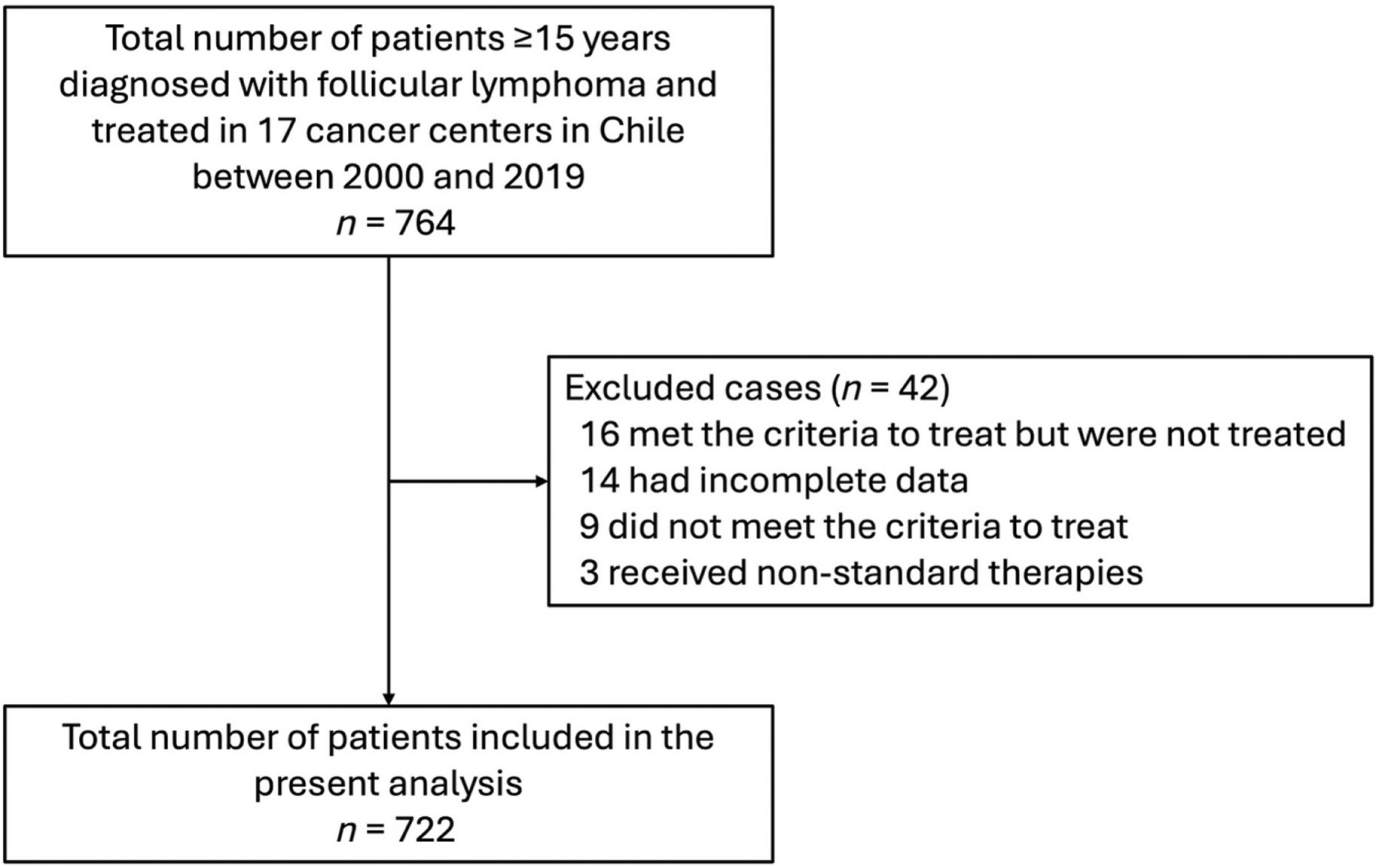
Flowchart of follicular lymphoma patients diagnosed between 2000 and 2019 in Chile.

Seven hundred and sixty‐four patients with FL were diagnosed between 2000 and 2019. Forty‐two were excluded from the analysis for different reasons (Figure 1). Seven hundred and twenty‐two patients were evaluated: Hospital del Salvador (*n* = 218), Instituto Nacional del Cáncer (*n* = 100), San Juan de Dios (*n* = 73), Sótero del Río (*n* = 68), Barros Luco (*n* = 51), San Borja (*n* = 40), Gustavo Fricke (*n* = 37), Valdivia (*n* = 30), Las Higueras (*n* = 28), Talca (*n* = 27), Arica (*n* = 20), Van Buren (*n* = 11), La Serena (*n* = 8), Temuco (*n* = 4), Osorno (*n* = 4), Antofagasta (*n* = 2), and Concepción (*n* = 1).

There was a female predominance of 1.7:1 (63%), the median age at diagnosis was 62 years (range, 15–93), and 412 cases (56%) were ≥60 years old. Histological grade was obtained in 217 cases (30%): grade I/II 164 (75%) and IIIA/B 54 (25%). The clinical stage distribution was stage I/IE 69 cases (9.5%), stage II/IIE 124 (17%), stage III 224 (31%), and stage IV 305 (42%). Extranodal sites included gastrointestinal, thyroid, subcutaneous tissue, and testicles, and 208 (68%) had bone marrow involvement. FLIPI score was available in 642 (89%) of patients; 238 (37%) had low, 219 (34%) had intermediate, and 185 (29%) had high‐risk disease (Table 1).

**TABLE 1:** Baseline characteristics of 722 patients with follicular lymphoma treated with chemotherapy, chemoimmunotherapy, and other schemes.

Variable	Total	Chemo^a^	CIT^b^	Other^c^	*p*	Missing
		*N* = 462 (64%)	*N* = 217 (30%)	*N* = 43 (6%)		
Age, median (range)	62 (18–93)	62 (15–89)	58 (23–87)	74 (19–93)	<0.001	0
Age >60 years	383 (53%)	249 (54%)	99 (46%)	35 (81%)		
Male sex	265 (37%)	176 (38%)	75 (35%)	14 (33%)	0.57	0
ECOG ≥2	129 (20%)	86 (21%)	38 (18%)	5 (16%)	0.66	67 (9%)
B symptoms	99 (14%)	58 (13%)	33 (15%)	8 (19%)	0.41	0
BM involved	208 (29%)	138 (30%)	61 (28%)	9 (21%)	0.45	0
Elevated LDH	172 (27%)	118 (29%)	37 (24%)	7 (20%)	0.22	86 (12%)
Stage III–IV	529 (73%)	336 (73%)	167 (77%)	26 (60%)	0.08	0
Hemoglobin <12 g/dL	184 (30%)	122 (32%)	50 (26%)	12 (34%)	0.35	110 (15%)
FLIPI score						
Low	238 (37%)	142 (35%)	81 (41%)	15 (43%)	0.56	80 (11%)
Intermediate	219 (34%)	145 (36%)	65 (33%)	9 (26%)		
High	185 (29%)	120 (29%)	54 (27%)	11 (31%)		

Abbreviations: ECOG, Eastern Cooperative Oncology Group; LDH, lactate dehydrogenases.

^a^ Includes CHOP, COP and CHOP/COP.

^b^ Includes R‐CHOP, R‐COP.

^c^ Includes CL, RT; FLIPI, follicular lymphoma international prognostic index.

### 3.2 Treatment

The median time to first treatment (TTFT) was 0.07 years (95% CI 0.06–0.08). The 1‐year TTFT rate was 96% (94–97), and the 5‐year TTFT rate was 99.4% (98.6–99.8).

Treatment was separated into three groups: 462 (64%) were treated with CT alone (CHOP, CHOP/COP, COP), 217 (30%) with CIT (R‐CHOP, R‐COP), and 43 (6%) with other regimes (CL, RT). The CT distribution was as follows: 314 (43%) received CHOP, and 83 (11%) CHOP/COP, COP 65 (9%). The CIT distribution was 111 (15%) received R‐COP, and 106 (15%) R‐CHOP. Other therapies included CL 35 (5%) and RT alone 8 (1%). Median number of cycles for CHOP was 6 (range, 1–8), CHOP/COP 12 (range, 6–12), COP 6 (range, 1–12), R‐CHOP 6 (range, 1–8), R‐COP 6 (range, 1–8), and CL 12 (range, 1–18). Rituximab maintenance was not used. The response of each chemotherapy regimen is presented in Table 2.

**TABLE 2:** Response rate of different regimens used to treat 722 patients with follicular lymphoma.

Regimen	CR	PR	NR	Total
CHOP	178 (57%)	92 (29%)	44 (14%)	314 (100%)
CHOP/COP	65 (78%)	12 (14%)	6 (7%)	83 (100%)
COP	32 (49%)	21 (32%)	12 (18%)	65 (100%)
R‐CHOP	69 (66%)	22 (21%)	14 (13%)	105 (100%)
R‐COP	80 (71%)	14 (13%)	18 (16%)	112 (100%)
Other	19 (44%)	19 (44%)	5 (11%)	43 (100%)

Abbreviations: CR, complete response; NR, no response (includes stable and progressive disease); PR, partial response.

Complete remission (CR) was obtained in 275/462 (60%) treated with CT, 149/217 (69%) treated with CIT, and 19/43 (44%) treated with other regimens (*p* < 0.001). Partial remission (PR) was obtained in 125 (27%), 36 (17%), and 19 (44%), respectively.

Of 217 cases with relapsed or refractory disease, 70 (32%) received FCM, 68 (31%) ESHAP, and the rest different schemes such as CHOP, R‐CHOP, R‐COP, CL, R‐bendamustine, or palliative care. Among the treated patients, 179 (82%) cases attained CR/PR.

### 3.3 Survival Analyses

The median follow‐up of the whole group was 15.1 years (95% CI 14.3–15.9), with a median of 17.1 years (95% CI 16.5–18.0) for the CT group and 10.9 years (95% CI 10.2–11.6) for CIT group (*p* < 0.001). Given a large number of censored events before the median was reached, the estimated median OS in the CIT group might be an underestimation.

In the univariate analysis, age >60, male sex, ECOG ≥2, B symptoms, BM involvement, elevated LDH, stage III/IV, hemoglobin <12 g/dL, and intermediate‐ and high‐risk FLIPI were associated with a statistically significant inferior OS. Using CIT (vs. CT) was the only variable associated with a superior OS (HR 0.55; 95% CI 0.43–0.70; *p* < 0.001). In a multivariate model, which did not include the FLIPI score and adjusted for age >60, male sex, ECOG ≥2, elevated LDH, stage III/IV, and hemoglobin <12 g/dL, using CIT (vs. CT) was an independent prognostic factor for a superior OS (HR 0.55; 95% CI 0.42–0.72; *p* < 0.001). Univariate and multivariate models are shown in Table 3.

**TABLE 3:** Cox proportional‐hazard regression analysis of different prognostic factors in 722 patients with follicular lymphoma.

Risk factor	Univariate analysis		Multivariate analysis^a^	
	HR (95% CI)	*p*	HR (95% CI)	*p*
Age (≥60 years)	2.27 (1.88–2.76)	<0.001	2.34 (1.86–2.93)	<0.001
Male sex	1.39 (1.15–1.68)	0.001	1.46 (1.18–1.81)	0.001
ECOG ≥2	2.06 (1.64–2.60)	<0.001	2.01 (1.55–2.60)	<0.001
B symptoms	1.44 (1.11–1.85)	0.006	1.13 (0.73–1.74)	0.58
Bone marrow involved	1.47 (1.21–1.79)	<0.001	0.88 (0.68–1.12)	0.29
Elevated LDH	1.65 (1.33–2.04)	<0.001	1.41 (1.12–1.78)	0.003
Stage III–IV	1.98 (1.57–2.49)	<0.001	1.65 (1.26–2.17)	<0.001
Hemoglobin <12 g/dL	1.94 (1.57–2.39)	<0.001	1.58 (1.26–1.98)	<0.001
1L treatment				
Chemo^b^	Ref.		Ref.	
CIT^c^	0.55 (0.43–0.70)	<0.001	0.55 (0.42–0.72)	<0.001
Other^d^	1.32 (0.93–1.88)	0.12	1.00 (0.65–1.55)	0.99
FLIPI score				
Low (0–1)	Ref.			
Intermediate (2)	2.12 (1.64–2.73)	<0.001		
High ≥3	3.61 (2.80–4.67)	<0.001		

Abbreviations: CI, confidence interval; CIT, chemoimmunotherapy; ECOG, Eastern Cooperative Oncology Group; FLIPI, follicular lymphoma international prognostic index; HR, hazard ratio; LDH, lactate dehydrogenase.

^a^ The FLIPI score was not included in the multivariate analysis.

^b^ Includes CHOP, COP, and CHOP/COP.

^c^ Includes R‐CHOP and R‐COP.

^d^ Includes chlorambucil and radiotherapy only.

**FIGURE 2:**
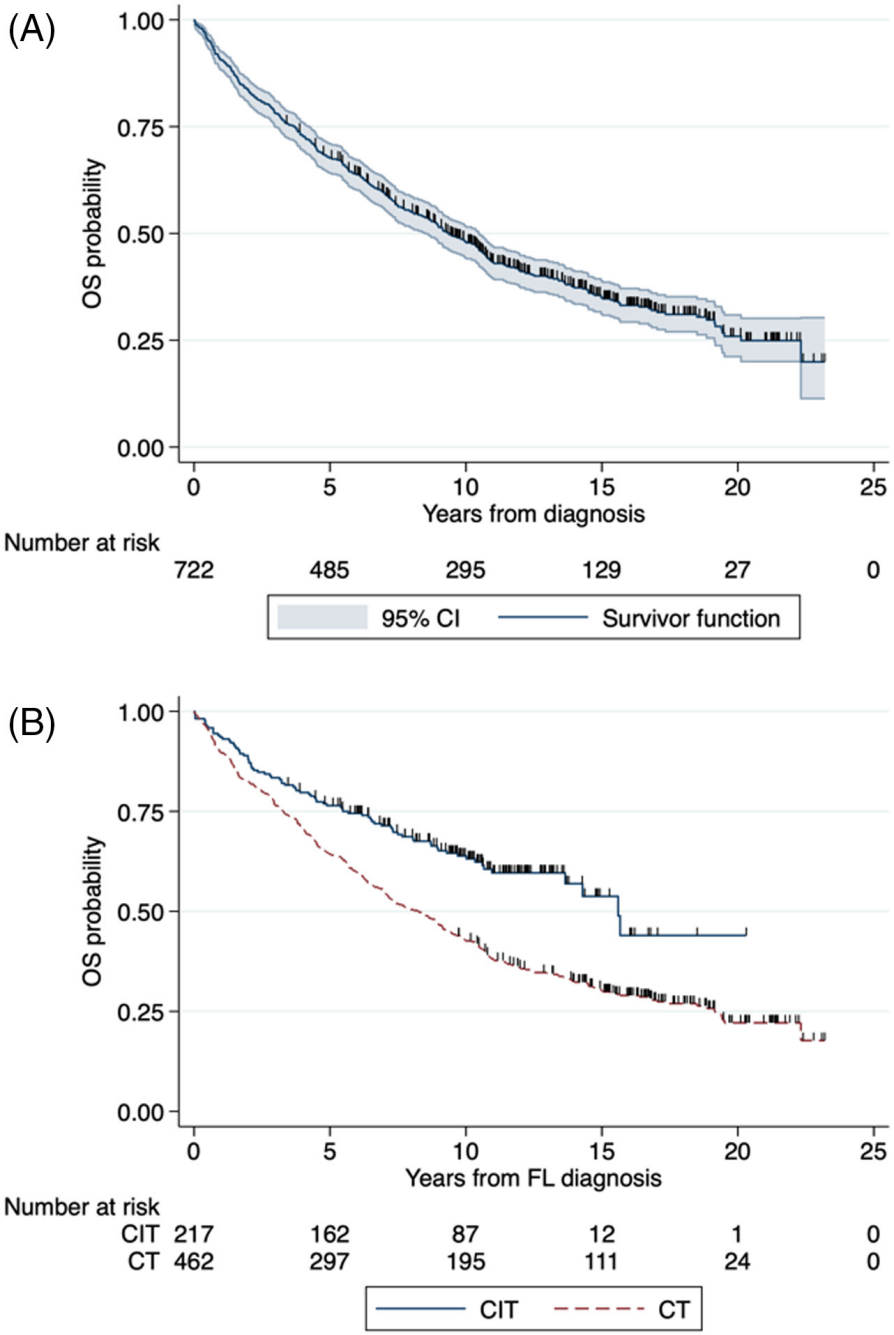
(A) Overall survival in all patients; and (B) according to frontline treatment.

The median OS for the whole group was 9.4 years (95% CI 8.5–10.5; Figure 2A), with a 10‐year OS rate of 48% (95% CI 44–52). The median OS for CT was 8.2 years (95% CI 7.0–9.2), with a 10‐year OS rate of 43% (95% CI 38–47). For CIT, the median OS was 15.6 years (95% CI 13.6‐not reached), with a 10‐year OS rate of 64% (95% CI 57–70). The difference in OS between CIT and CT was statistically significant (*p* < 0.001; Figure 2B). For other regimens, the median OS was 6.9 years (95% CI 3.1–9.3), with a 10‐year OS rate of 34% (95% CI 20–48).

**FIGURE 3:**
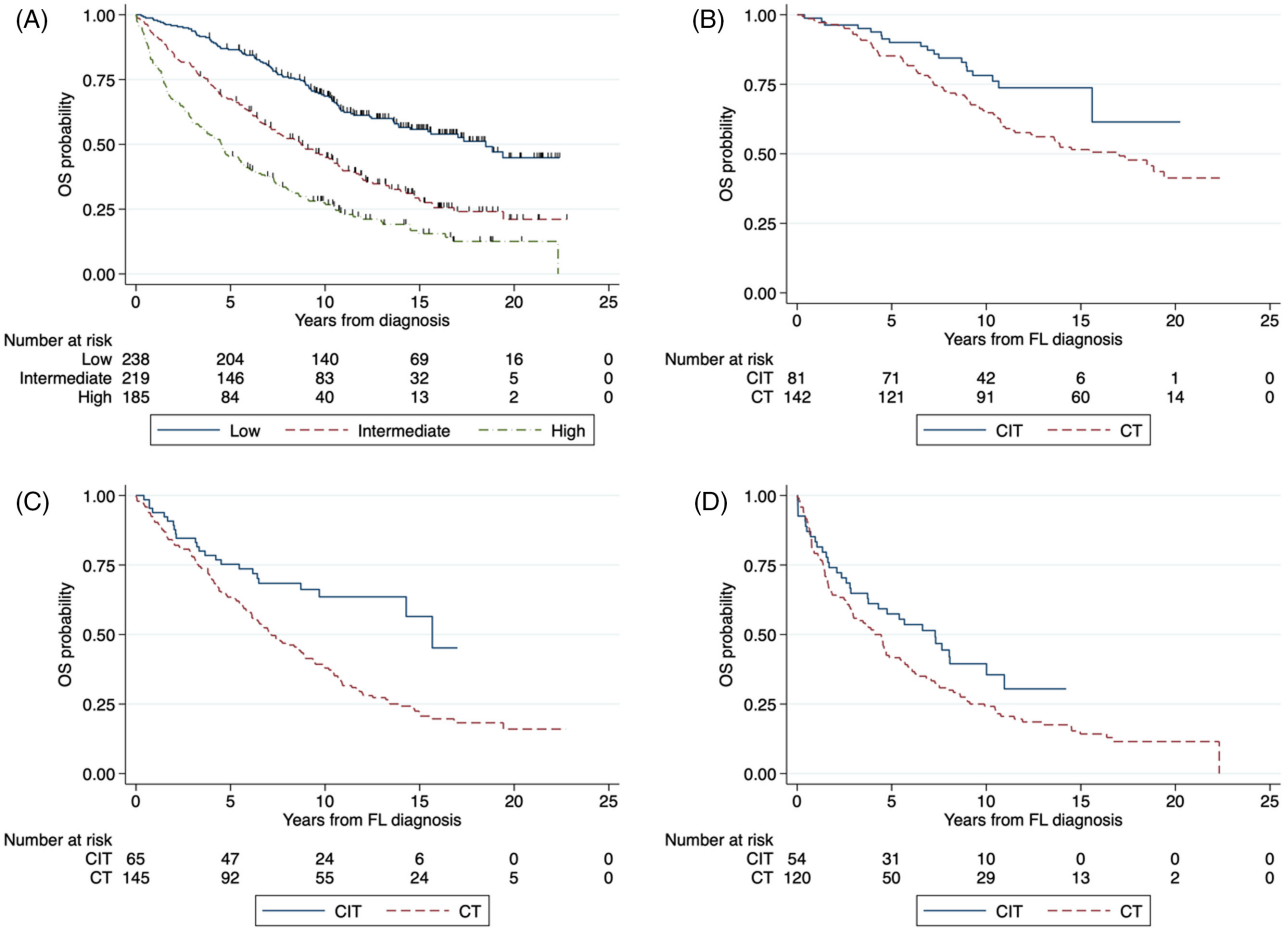
(A) Overall survival according to FLIPI score; (B) according to CIT versus CT in low IPI; (C) according to CIT versus CT in intermediate IPI; and according to CIT versus CT in high IPI (D).

There was a significant difference in OS according to the FLIPI score. The median OS for low risk was 18.5 (95% CI 13.9‐NR), for intermediate 8.7 (95% CI 7.0–10.5), and for high risk 4.5 years (95% CI 3.3–5.6). The 10‐year OS rate for low risk was 69% (95% CI 62–74), for intermediate 45% (95% CI 38–52), and for high risk 28% (95% CI 21–34) (*p* < 0.001; Figure 3A). In patients with low FLIPI, CIT was associated with a higher 10‐year OS rate of 78% (95% CI 67–86) versus CT at 65% (95% CI 56–72) (*p* = 0.02; Figure 3B). In patients with intermediate FLIPI, CIT was associated with a higher 10‐year OS rate of 64% (95% CI 50–75) versus CT at 38% (95% CI 30–46) (*p* < 0.001; Figure 3C). In patients with high FLIPI, CIT was associated with a numerically higher 10‐year OS rate at 39% (95% CI 26–53) versus CT at 24% (95% CI 17–32) (*p* = 0.07; Figure 3D).

**FIGURE 4:**
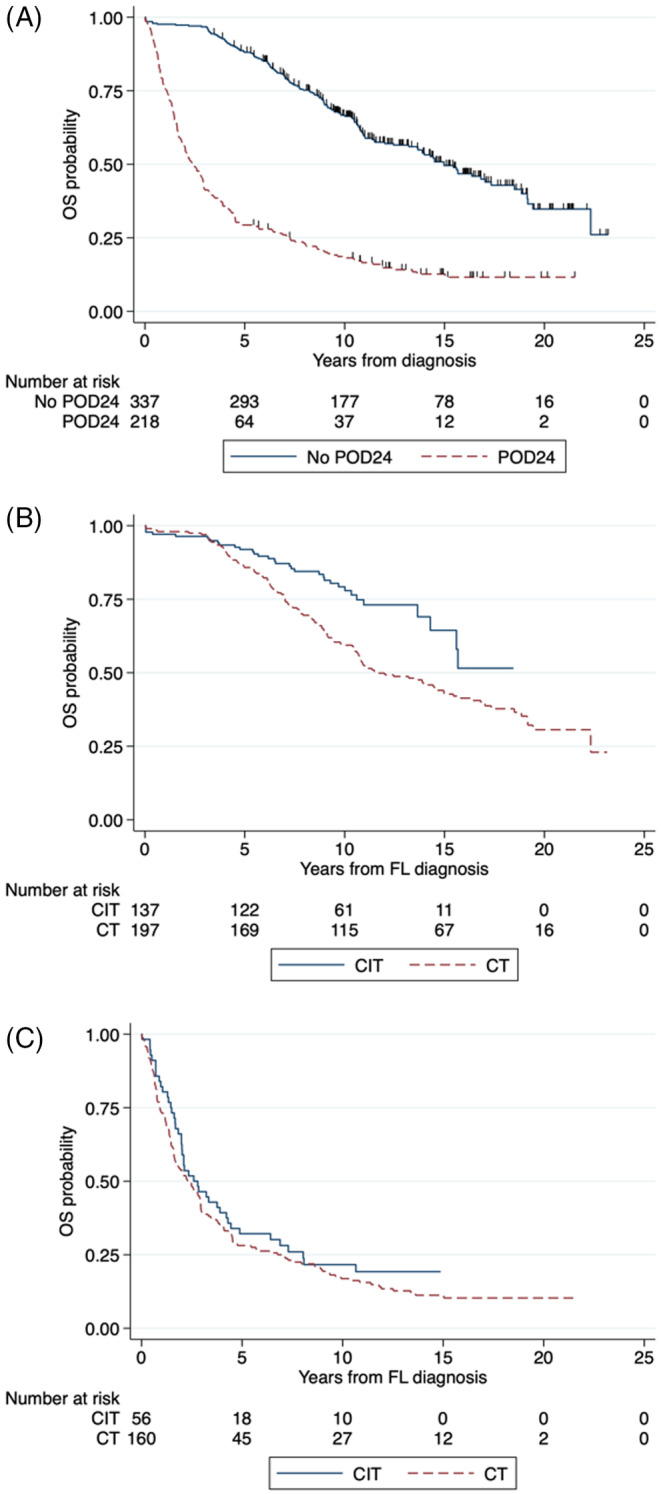
(A) Overall survival according to POD2; (B) according to CIT versus CT in patients who did not experience POD24; and (C) according to CIT versus CT in patients who experienced POD24.

POD24 was analyzed in 555 (77%) cases. The median OS for the no POD24 group was 15.0 years (95% CI 13.2–17.3), while for POD24 was 2.4 (95% CI 2.0–2.9). The 10‐year OS rate for no POD24 was 67% (95% CI 61–72), while for POD24 was 18% (95% CI 13–24) (*p* < 0.001; Figure 4A). CIT was associated with a higher 10‐year OS rate than CT in patients who did not experience POD24 (79%, 95% CI 71–86, vs. 59%, 95% CI 52–66; *p* < 0.001; Figure 4B). There was no difference in the 10‐year OS rate between CIT and CT in patients who experienced POD24 (*p* = 0.28; Figure 4C).

In 24 (3%) patients, a transformation to a DLBCL was confirmed, with a median time to transformation of 7.2 years (range, 1–10).

### 3.4 Cause of Death

Of 452 patients who have died, the cause of death (COD) was known in 447 (99%). The most common was lymphoma, 330 (75%). The second was treatment complications, 43 (10%), mainly infections, half of them in patients >70 years old and 8 from COVID‐19 pneumonia. Secondary malignancies were the third cause, 34 (8%), which included solid tumors 25 (gastrointestinal 11, genitourinary 6, lung 3, other 6) and hematological neoplasia 9 (SMD/AML 7, ALL and ATL, one each). Cardiovascular disease (CVD) was the fourth cause, 23 (5%), and included heart failure, stroke, acute myocardial infarction, pulmonary thromboembolism and ruptured aortic aneurysm. Seventeen patients (4%) had other COD: renal failure 6, lung fibrosis 3, diabetes mellitus 3, digestive hemorrhage 3, suicide 1, amyotrophic lateral sclerosis 1 (Table 4).

**TABLE 4:** Causes of death of 447 patients with follicular lymphoma.

Cause	Total	Chemo^a^	CIT^b^	Other^c^
	*N* = 447 (100%)	*N* = 330 (74%)	*N* = 83 (18.5%)	*N* = 34 (7.5)
Lymphoma	330 (75)	242	60	28
Infection complication	43 (10)	33	9	1
Secondary malignancies^a^	34 (8)	25	8	1
Solid	25	18	6	1
Hematologic	9	7	2	0
Cardiovascular	23 (5)	18	3	2
Other causes^d^	17 (4)	12	3	2
Unknown^e^		5 (1)		

^a^ Includes CHOP, COP, and CHOP/COP.

^b^ Includes R‐CHOP and R‐COP.

^c^ Includes chlorambucil.

^d^ Other causes, described in results.

^e^ Not included in the percentages.

If we analyze the COD of those patients who died within the first 5 years of diagnosis and compare it with those who survived more than 5, 10, or 15 years, lymphoma decreased progressively, while infection complications, secondary malignancies, and CVD increased >4‐fold over time (Table 5).

**TABLE 5:** Cause of death of 442 patients with follicular lymphoma surviving different periods of time.

Variable	Total	<5 years OS	≥5 years OS	≥10 years OS	≥15 years OS
		*N* = 233	*N* = 214	*N* = 84	*N* = 19
Lymphoma	330 (75%)	195 (84%)	135 (63%)	35 (42%)	5 (26%)
Infection complication	44 (10%)	13 (6%)	30 (14%)	19 (23%)	6 (32%)
Secondary malignancies	35 (8%)	9 (4%)	25 (12%)	14 (17%)	4 (21%)
Cardiovascular diseases	22 (5%)	8 (3%)	14 (6%)	10 (12%)	4 (21%)
Other causes	11 (2%)	8 (3%)	10 (5%)	6 (6%)	0 (0%)

## 4 Discussion

The present study shows the evaluation from the largest cohort of FL in Chile, treated in the Public Health System with a long follow‐up of 15 years and diagnosed over 20 years. We analyzed 722 patients and showed that chemoimmunotherapy (CIT) significantly improved survival, compared with chemotherapy (CT) alone, with a 10‐year OS rate of 64% versus 43%, respectively. FLIPI and POD24 were important prognostic factors for survival.

Multiple randomized trials have demonstrated the benefit of adding rituximab to combination chemotherapy for the initial treatment of advanced‐stage FL [9, 10, 11, 12]. Data from the Swedish Lymphoma Registry from 2003 to 2010 reported a 10‐year OS rate of 79% [13]. This population‐based lymphoma registry found that survival increased progressively from 2000 to 2010 since rituximab was introduced in 2003. Survival was superior in regions where first‐line rituximab was quickly adopted and inferior in regions where rituximab was slowly adopted.

A nationwide population‐based study from the Netherlands showed 5‐year relative survival from 2009 to 2016 stage III/IV to be 90%, 83%, and 68% by age groups 18–60, 61–70, and ≥70, respectively [14]. Analysis of French and US cohorts also reported improved OS in the rituximab era, with a 10‐year OS rate of approximately 80% [21, 22]. In a study by Rajamäki et al. [23] with 749 patients from Finland and Spain, the estimated 10‐year OS rate was somewhat lower at 72%. Our results appear inferior to the above observations, even after introducing CIT. One of the explanations may be that first‐line rituximab was adopted at different speeds in different regions, considering this is a 4200 km long country. The quickness to treat infections may also differ from center to center, considering that infections were the second most common COD after lymphoma. Half of these patients were older, >70 years old, and eight deaths were due to COVID‐19 pneumonia during the pandemic.

FLIPI has remained a useful prognostic model even in the rituximab era [5, 24, 25]. Our study also found that FLIPI was a highly significant tool that predicted clinical outcomes with 10‐year OS of 69% in the low risk compared to 28% in the high‐risk group. The same was observed with the negative impact of POD24, which has become a surrogate for poor outcomes [8, 26, 27, 28]. The 10‐year OS rate for no POD24 of 67% (95% CI 61–72), was significantly better compared to those who experience POD24, only 18% (95% CI 13–24) (*p* < 0.001). Our results are comparable to those described by Casulo et al. [27], who reported a 5‐year OS of 90% of no POD24, contrasting with 29% in those who experienced POD24. There was no difference in the 10‐year OS rate between CIT and CT in patients who experienced POD24 (*p* = 0.28), likely a reflection of more aggressive disease with poor outcomes regardless of the frontline treatment used. There have been efforts to identify predictors of POD24, but the precise mechanisms underlying early progression are still undefined.

Analyzing the COD, lymphoma was the leading cause. Similar results have been reported elsewhere [29, 30, 31]. Regarding secondary malignancies, our study showed 8%, which is similar to that published by Sacchi et al., [32], who reported 6.9%, primarily solid tumors, and increases over time. Others have reported a significant incidence of fatal secondary myeloid neoplasia in patients who received radioimmunotherapy or a fludarabine‐mitoxantrone‐based regimen [22]. Cardiovascular disease also increased over time. This event was not mentioned in the above studies, even though the age of the population may well explain it.

Transformation to aggressive lymphoma accounts for a large portion of lymphoma‐related mortality. The risk of transformation is reported to be around 20% at 5 years and 30% at 10 years [33], while others [31] have reported lower numbers (≈12%). The lower number in our series compared with the literature, 3.3%, is probably due to low suspicion and biopsy taken.

The great predominance of women in this study was striking, 63%, compared to the male gender, even greater than in previous NHL studies from Chile (54% and 55%) [3, 34]. In the epidemiological study of NHL in Chile, we saw women predominance in DLBC (51%), FL (61%) and mainly in marginal zone lymphoma (74%). It is striking to see that in the Hungria study [35], including over 5000 patients with hematologic malignancies in Latin America, female predominance was seen in DLBCL in Brazil (57%) and Mexico (52%). Even more, regarding FL, women predominate in every country, 55% as a whole, especially in Brazil (65%), Argentina (56%), and Chile (53%). So, this is a consistent finding, in contrast to what is described almost everywhere, with male gender predominance in all subtypes of lymphomas [36, 37]. Most notably, the female gender predominate also in multiple myeloma in Chile [38, 39], unlike what is described in the literature [40, 41]. The reasons for this finding need to be clarified. It may be partly due to women being more likely to seek medical assistance or an actual biological difference in Latin American patients.

There is limited information regarding FL in Latin American countries [3, 35, 42, 43]. The information available is focused mainly on epidemiology. In the Laurini comparison [42], Chile had a higher incidence of FL, like Argentina, compared to other countries like Brazil, Guatemala, or Peru, as also reported in the Villela et al. study [43]. Similarly, the median age in Chile is higher than in the other countries studied, in concordance with the higher Human Development Index (life expectancy, literacy rate, and GDP per capita) [44].

The main strength of our study is the large cohort of FL patients and an extended follow‐up, as well as the reliability of the COD data in Chile, known in almost 100% of patients. To our knowledge, studies of similar size in Hispanic populations are lacking. The limitations of our study include a lack of progression/relapse date in many cases, so we could not calculate PFS. Since it is a registry‐based study, there could have been biases in the quality of the data registered. Also, there were missing data, which was addressed with listwise case deletion, a reasonable approach given the cohort size and the data missing at random. Finally, this study comprised Chilean patients, which might limit the applicability of our findings in non‐Hispanic population.

In conclusion, adding rituximab to chemotherapy in FL was associated with significant survival improvement in patients treated in the Public Health System in Chile. We also validated FLIPI and POD24 as prognostic factors. We need to improve the outcome of FL by incorporating more efficacious second‐line treatments.

## Author Contributions

**María Elena Cabrera:** conceptualization, methodology, investigation, validation, formal analysis, supervision, writing–original draft, writing–review and editing. **Camila Peña:** formal analysis, writing–original draft, investigation, writing–review and editing. **Valeska Vega:** investigation, writing–review and editing, writing–original draft. **Hernán Rojas:** investigation, writing–original draft, writing–review and editing. **Alvaro Pizarro:** investigation, writing–original draft, writing–review and editing. **Christine Rojas:** investigation, writing–original draft, writing–review and editing. **Susana Calderon:** investigation, writing–original draft, writing–review and editing. **Jacqueline Oliva:** investigation, writing–original draft, writing–review and editing. **Cecilia Hales:** investigation, writing–original draft, writing–review and editing. **Bernardita Rojas:** investigation, writing–original draft, writing–review and editing. **Marvila Intriago:** investigation, writing–original draft, writing–review and editing. **Marisa Capurro:** investigation, writing–original draft, writing–review and editing. **M. Luisa Gonzalez:** investigation, writing–original draft, writing–review and editing. **Jorge J. Castillo:** methodology, software, data curation, validation, formal analysis, supervision, writing–original draft, writing–review and editing.

## Conflicts of Interest

J.J.C. reports research funds and/or consulting fees from AbbVie, AstraZeneca, Beigene, Cellectar, Janssen, Kite, LOXO, Mustang Bio, and Pharmacyclics. All other authors have no conflict of interest to report.

## Data Availability

The data that support the findings of this study are available from the corresponding author upon reasonable request.
